# Establishing and sustaining authentic organizational partnerships in childhood disability research: lessons learned

**DOI:** 10.1186/s40900-023-00466-x

**Published:** 2023-07-20

**Authors:** Patrick G. McPhee, Kinga Pozniak, Mary A. Khetani, Wenonah Campbell, Leah Dix, Michelle Phoenix

**Affiliations:** 1 Department of Psychiatry and Behavioural Neurosciences, Health Sciences Centre, 3N26, McMaster University, 1280 Main Street West, Hamilton, ON L8S 4K1 Canada; 2 Offord Centre for Child Studies, McMaster University, Hamilton, ON Canada; 3 School of Rehabilitation Science, McMaster University, Hamilton, ON Canada; 4 CanChild Centre for Childhood Disability Research, McMaster University, Hamilton, ON Canada; 5 Department of Occupational Therapy, University of Illinois Chicago, Chicago, IL USA

**Keywords:** Partnership, Organization, Childhood disability, Auto-ethnography

## Abstract

There is an increased interest from both researchers and knowledge users to partner in research to generate meaningful research ideas, implement research projects, and disseminate research findings. There is accumulating research evidence to suggest the benefits of engaging children/youth with disabilities and their parents/families in research partnerships; however, less is known about the benefits of, and challenges to, engaging organizations as partners in research. The purpose of this commentary is to reflect on successful organizational partnership experiences from the perspectives of researchers at an internationally-recognized childhood disability research centre (*CanChild*), and to identify and share key ingredients for developing partnerships between organizations and academic institutions. A companion study is underway to examine partnership experiences with *CanChild* from the partners’ perspective. Four *CanChild* researchers and two co-facilitators participated in a collaborative auto-ethnography approach to share experiences with organizational research partnerships and to reflect, interpret, and synthesize common themes and lessons learned. The researchers and facilitators met virtually via Zoom for 105 min. Researchers were asked to discuss the following: the formation of their organizational partnerships; if/how partnerships evolved over time; if/how partnerships were sustained; and lessons learned about benefits and challenges to building research partnerships with organizations. The meeting was recorded, transcribed verbatim, and analyzed by the facilitators to identify and synthesize common experiences and reflections. Multiple rounds of asynchronous reflection and feedback supported refinement of the final set of analytic themes. Researchers agreed that partnerships with organizations should be formed through a mutual interest, and that partnerships evolved by branching to include new organizations and researchers, while also involving trainees. Researchers identified the importance of defining roles and responsibilities of key individuals within each partnering group to sustain the partnership. Lessons learned from organizational partnerships included reciprocity between the partnering organization and academic institution, leveraging small pockets of funds to sustain a partnership over time, and building a strong rapport with individuals in a partnership. This commentary summarized lessons-learned and provided recommendations for researchers and organizations to consider when forming, growing, and sustaining research partnerships over time.

## Abstract (plain language summary)

Researchers and people who use research findings are partnering to create research projects and share results. There are examples of children with disabilities and their families participating in research partnerships, but less is known about the involvement of healthcare organizations and community organizations as research partners. The purpose of this article is to share successful examples of partnership between organizations and a childhood disability research centre from the perspective of researchers. Four researchers and two facilitators met to reflect on their experiences with organizational research partnerships. They met online for 105 min using Zoom software. The researchers were asked to talk about how their partnerships with organizations were formed, how they grew over time, and how they were maintained. The meeting was recorded, and the facilitators took the researchers’ experiences and summarized them into common messages. Everyone then read the summary on their own and added their ideas. This happened three different times until everyone agreed on one set of ideas. The researchers agreed that partnerships with organizations should be formed through common goals, that they should grow to include new partners and junior researchers, and that clear roles and responsibilities were needed to keep the partnership going. The experiences shared in this article are valuable to other researchers and organizations that are interested in forming research partnerships.

## Background

Increasingly, health care organizations, funding bodies, policy-makers, and professional organizations are interested in partnering with diverse knowledge users in research. Many well established research approaches or methodologies include aspects of partnership, such as integrated knowledge translation (iKT), collaborative research, community-engaged research, community-based participatory research, or co-production of knowledge [1]. In Canada, iKT is the term adopted by the Canadian Institutes of Health Research [2]. The foundation of iKT is built on authentic scholarly partnerships between knowledge users and researchers, to rigorously generate and share knowledge for practice. Knowledge users are those who are actively involved in the process of producing knowledge from a study and who may benefit or be affected by the research [3]. A Knowledge User is an individual who is likely to utilize research results to make informed decisions about health policies, programs, and/or practices. Knowledge users who bring contextual and lived experience to research teams may include, but are not limited to, patients and patients’ families, children/youth and their families, clinicians, organizations, and policy makers [2]. Through research partnerships, knowledge users can contribute their perspectives and experiences by: 1) generating research question(s) that are meaningful to practice or policy, 2) refining the design of a study or project (including selection of the methodology), 3) implementing the study or project (including data collection and outcome measure selection or development), and/or 4) interpreting and tailoring dissemination of research findings [4]. Researchers tend to hold academic degrees and a position that allows them to design and conduct research. Researchers can choose to engage one or more knowledge user groups in a single study and/or across multiple studies in a programmatic line of inquiry. The definition of research partnership is intentionally fluid because the context in which research is conducted and the people involved may influence which knowledge users are engaged and the nature of their engagement.

There is emerging evidence about involving children/youth with disabilities and/or their families as partners in childhood disability research, including a systematic review of 22 articles that identified benefits and challenges of including children and young people with disabilities as partners in research [5]. Benefits for children/youth with disabilities as knowledge users included increased self-confidence, self-esteem, and independence. Conversely, challenges included finding sufficient time, communication, planning and financial and personnel resources [5]. Recent research by some of our co-authors identified key ingredients for successful parent-research partnerships, such as communication, identifying roles and expectations, self-reflection, and resources (e.g., funding) [6, 7]. Relative to the literature on engaging individual children/youth and their families, less is known about the benefits of, and challenges to, engaging health care and community organizations as partners in research. An organizational partnership can be defined as a collaborative research activity involving at least one researcher (e.g., individual associated with an academic or research institution) and any organizational stakeholder (e.g., health care leader of an organization) actively engaged in any part of the research process [8, 9]. Research collaborations involving knowledge users within organizations are pivotal to developing data-driven solutions for service access, use, and quality.

*CanChild* Centre for Childhood Disability Research (hereafter referred to as *CanChild*) was created in 1989 and is located at McMaster University in Hamilton, Ontario, Canada. It is a research centre comprised of local, national, and international students, researchers, and partners who collaborate in conducting childhood disability research. *CanChild* researchers from different academic institutions have historically engaged in research partnerships with diverse knowledge users, ranging from individual children and youth with disabilities and/or their families, to organizational stakeholders such as service providers, program leadership, and/or policy makers. *CanChild* researchers have contributed to best practice guidelines for engaging various stakeholder groups in research and knowledge translation [10, 11] and have identified growing partnerships as a key focal area for knowledge translation advancement in their 2020–2025 strategic plan [12].

The purpose of this paper is to examine successful organizational partnership experiences at *CanChild* from the perspectives of researchers and identify key ingredients for fostering research partnerships between organizational and academic institutions. While our group has studied research partnership trajectories with children, youth, and families [6, 13], we have not previously studied our organizational research partnerships, including how these types of partnerships were formed, maintained, and have evolved over time. Understanding researchers’ lessons-learned and providing recommendations that other researchers and organizations can consider will be valuable for future partnership research. A companion study is underway to examine partnership experiences with *CanChild* from the partners’ perspectives and is expected to provide further guidance.

## Reflecting together to understand organizational partnerships

*CanChild* researchers took part in a collaborative auto-ethnography approach to gather descriptions of their experiences with organizational research partnerships and to reflect, interpret, and generate common themes and lessons learned. Collaborative auto-ethnography is a form of qualitative research in which authors use self-reflection and writing to explore anecdotal and personal experiences and connect these autobiographical stories to wider meanings and understandings [14]. It “brings together the self-reflection associated with ethnography, and multi-subjectivity associated with collaboration” [14]. Collaborative auto-ethnography is carried out collectively by a group of researchers who work together to collect, analyze and interpret their own combined data in order to gain a meaningful understanding of the phenomena reflected in their individual accounts [15]. Researchers can employ a range of data collection methods, including interviewing each other, analyzing each other’s reflections, or collecting archival data about one another. Stage 1 in this project was completed as part of a strategic planning process in *CanChild* in which approximately 15 trainees, local and international researchers discussed how best to advance “Intentional Collaboration”. These discussions identified organizational partnerships as a strength at *CanChild* and a need to further develop, learn from, and share examples from well-established organizational partnerships. Stage 2 included selection of the example teams and projects and their participation in a focus group (i.e., collaborative auto-ethnography). Stage 3 included three rounds of asynchronous reflection and feedback on the interim analytic themes.

Researchers in the present study included those with previously published and ongoing research partnerships with organizations [16–18]. We sought to reflect on research projects that represent a breadth of organizational partnerships according to the: 1) number of organizational partners, 2) types of organizational partners (e.g., clinical organization, family network, government representatives and policy makers, school, community child care, and rehabilitation networks), and 3) duration of the partnership. These included the following research projects: Readiness Support Project [19]; Patient-Reported Outcomes for Strengthening Partnership in Early Intervention Care Teams (PROSPECT) [16, 20, 21]; and Partnering for Change (P4C) [18, 22]. Brief descriptions of each project or study can be found in Table 1.

**Table 1:** Research projects involving organizational partnership

Study	Year(s)	Description	Organizational Partner(s)
Readiness Support Project	2013-present	The Readiness Support Project grew from a program of research conducted in partnership with KidsAbility, a Children’s Treatment Centre in Ontario Canada. This program aims to improve attendance and engagement in children’s rehabilitation services by co-developing, implementing and evaluating changes in service delivery that reduce barriers to service use and improve equity in service delivery	The organizational partners included a parent, who facilitated communication and involvement with other parents as needed (e.g., document review), multiple clinicians, and an organizational manager who fulfilled the role of knowledge broker
Patient-Reported Outcomes for Strengthening Partnership on Early Intervention Care Teams (PROSPECT) Project	2016-present	Patient-Reported Outcomes for Strengthening Partnership in Early intervention Care Teams (PROSPECT) is the latest phase of work for an organizational research partnership that has focused on improving family-centered and participation-focused service design and research for quality improvement of early intervention services. PROSPECT has examined the effectiveness and implementation of the Participation and Environment Measure (PEM) electronic assessment, when paired with a program-specific decision support tool, for strengthening service design and quality program improvement targeting children 0–3 years with developmental needs and their families. An extension of PROSPECT is examining the effectiveness and implementation of the PEM electronic assessment together with its companion goal setting application, for a similar purpose	This study involves partnership with one community organization
Partnering for Change	2008-present	Partnering for Change (P4C) is the name of both an innovative, evidence-informed model that guides the delivery of tiered rehabilitation services in schools and the program of research focused on its implementation and evaluation. Created by researchers at *CanChild*, P4C was developed and tested using a participatory action research process involving key stakeholders from government, health care decision-makers, occupational therapists, service provider organizations, schools, and families. The goals of P4C include early identification of children with special needs; building capacity of educators and families to understand and manage children’s needs; preventing secondary consequences associated with children’s unmet needs; and improving children’s ability to participate successfully at school	Over the lifespan of the program of research, the P4C team has partnered with 13 different health care organizations who provide occupational therapy services in schools, 39 occupational therapists, 13 school districts, 60 schools as well as the three government ministries that fund the services and the research. Each organization identifies designated representatives who collaborate with the research team via working groups and steering committees

To facilitate discussions of these experiences, researchers were asked about how their organizational partnerships were formed; if (and how) the partnerships evolved over time; if (and how) the partnerships were sustained over time; lesson(s) learned about the benefits and challenges to building their research partnerships; and recommendations for other childhood disability researchers and scientists interested in partnering with organizations. Researchers were provided with these questions prior to meeting in a virtual setting to prepare their thoughts.

Four researchers and two co-facilitators met in a virtual setting using Zoom teleconference software (2023 Zoom Video Communications, Inc.) for 105 min. Each researcher first offered their reflections to each of the above questions and were afforded chances to ask each other follow-up questions to identify commonalities and differences in their experiences. The virtual meeting was recorded and transcribed verbatim. PGM and KP as co-facilitators, reviewed the transcriptions, collaboratively synthesized these experiences, and drafted the key ideas into manuscript form. Three iterations of the findings and manuscript were reviewed and revised by all researchers who participated in the videoconference to ensure that the information presented reflected their experiences, selected their most powerful examples, considered the diversity in exemplars provided, and communicated asynchronously using the comment and reply functions in the written document.

The discussion that follows describes a synthesized reflection of different organizational partnership experiences from researchers within *CanChild*, shared lessons learned, and provides recommendations for future research in this area. This article aims to present a collective voice of researcher experiences with organizational partnerships, while acknowledging the individual reflections of each researcher on each project or study. As collaborative auto-ethnography is a qualitative research design, we present our discussions and recommendations with the intent of providing our collective reflections and examples that may offer guidance for researchers who wish to conduct research in partnership with organizations [23].

## How partnerships were formed

All of the *CanChild* researchers who took part agreed that it was valuable for organizations and researchers to initiate partnerships through a shared interest in a topic or research question. A common starting point was perceived to be mutual interest from both the organizational and research institutions in developing a solution for a clinical challenge. For example, in P4C, a leader in a healthcare organization responsible for delivering school-based occupational therapy (OT) services, approached researchers to investigate service delivery issues (i.e., high wait lists for school-based occupational therapy services). Likewise, the Readiness Support Project was initially started at KidsAbility (a local organization delivering rehabilitation services to children [Table 1]) because the researcher (MP) worked clinically in the organization and had discussed the problem of missed appointments with clinicians, managers and leadership who wanted to revise models of service delivery and policies and to better support families and clinicians. These researchers also agreed that partnerships were formed by leveraging pre-existing relationships. However, it was noted that researchers did not necessarily need to have all partnerships in place when beginning their research, and they could leverage their existing partnerships to extend to new people or organizations.

## How the partnerships evolved

Researchers reflected on the opportunities for growing partnerships through linking in new partners and onboarding trainees. In PROSPECT, there was early opportunity to grow their new organizational partnership while partnering to solve a clinical challenge in a specific geographical locale. The researcher (MK) received initial funding as they were relocating to a new institution and their partner organization was initiating adoption of statewide changes impacting their current workflow. As a result of these major changes, they chose to “fail forward” together by experimenting with how to best conduct research together (e.g., failing with use of provider training to recruit and retain participants and therefore pivoting to a peer mentoring approach instead to move forward) and negotiating the parameters of their partnership (e.g., failing to retain providers when relying on authored refereed publications and presentations and therefore pivoting to co-producing a podcast instead). They created a new research group to recruit and retain organizational partners and co-author products, and they created norms and mechanisms to partner with *CanChild* researchers across multiple institutions and onboard trainees in a new academic institution and geographic locale. Trainees were able to advance partnership growth when they brought new partnership ideas to the researcher or when the researchers extended their existing partnerships to include trainees [20, 21]. An example of this was an extension of the Readiness Support Project where a trainee was added to the team who was a clinician at the partner organization and had an interest in investigating telerehabilitation services. By joining the team, she was able to expand the scope of the project. She also invited new team members from the organization that included parent partners, the organization’s research and innovation team members, and clinicians. Additionally, ongoing partnerships within *CanChild* led to extended partnerships between researchers; for example, the Principal Investigators of the P4C and Readiness Support Projects supported each other’s growth through introduction to new study partners and opportunities. This underscored the value of a (*CanChild*) research centre’s infrastructure to further partnerships.

## Maintaining partnerships

Individuals within organizational partnerships were identified by researchers as key players in the initiation and sustainability of partnerships. Layers within the partnership indicated the importance of multiple levels of engagement between researchers and organizational partners, and the roles and responsibilities of individuals within each partnering group. These roles and responsibilities included representation from each partner, and how every partner would like to be involved in each stage of the project, to ensure ongoing engagement and facilitation towards a shared end goal. For example, to support continued engagement, the P4C project developed and implemented a terms of reference guide to provide information about who is involved in the partnership, their mandate, and their roles and responsibilities. Similarly, PROSPECT implemented a dissemination guide at the start of each phase of work, to map each partner to their credited roles for contributing to research products. Across researchers, each partnership that was described grew over time, with new connections made with trainees, other *CanChild* colleagues, and/or external community organization colleagues, allowing the research to expand with increased reach and impact.

## Lessons learned and recommendations

The researchers identified three important lessons learned and recommendations for forming, evolving, and maintaining partnerships. Three illustrative quotes were selected and agreed upon by the researchers that named and captured the essence of these lessons. These are presented below.

“What do we offer to our partners in addition to thinking about what they offer back to us?” (MP)

A reciprocal relationship between an academic institution and a partnering organization is fundamental for establishing a foundation from which partnerships can strengthen and evolve [24]. In our (researcher) experience, a shared commitment to working and learning from one another suggests that partners will be invested, engaged, and encouraged to work collaboratively to achieve the goal(s) of the partnership. Sharing perspectives and expertise from stakeholders at the onset of a partnership ensures that opinions and desires were valued and would be included within the project. In Readiness Support, members of the research team worked with families, clinicians, and management to create new organizational policies to support families’ attendance and engagement in services. Even after the project ended, the researcher worked with the clinical team to revise the program, develop training for staff, and share evaluation findings. Reciprocity might also be offered through the experiences of organizations involved in the partnership. In academic/community partnerships, researchers benefited from hearing the insights and feedback from the community members that allowed them to challenge their own assumptions [25]. Being cognizant of the value of reciprocity in partnerships might ensure that all parties are satisfied, can deepen their learning, and will continue the partnership in the future.

“Build habits for being a good steward of smaller pockets of funds” (MK)

It is important to recognize and understand, early on, that it takes resources to build and sustain partnerships over time. We therefore acknowledge the importance of fully delivering on smaller initial investments in the partnership (e.g., seed-funding, in-kind contributions, or pilot grants), and establishing habits for negotiating risk while producing on project deliverables with ‘shoestring’ budgets, as they can reinforce creativity and accountability in the partnership as needed to seek and secure larger investments and rewards. A literature review on creating successful partnerships identified critical success factors for partnerships, which included sufficient funds, staffing personnel, materials and time, and skilled leadership [26]. Notwithstanding the importance of initial financial commitments to a partnership, we, as researchers, recommend reflecting early on in a partnership to allow those involved to understand what is working, what is not, and what might be missing in the partnership. This can be important for both the sustainability and evolution of partnership over time.

“People value the relationships and how they feel engaging in the partnership” (WC and LD)

Regardless of whether a program of research engages in a partnership with a single organization or multiple organizations, it is paramount to appreciate the quality and the authenticity that individuals and groups bring to the partnership. Researchers emphasized the importance of investing time and effort in establishing personal relationships with partners to make the partnership truly authentic. Building a rapport with partners that is authentic can lead to multiple partnerships in the future, with mutual benefits [27]. In P4C, the research team delivered presentations to school-board partners on topics of interest that addressed the partner’s requests for information sharing. By routinely meeting these requests, the team built rapport and established relationships necessary to proceed with the research. Institutions and organizations should strive towards establishing partnerships where all parties are engaged, feel valued, and work towards common goals [22].

### Limitations

Although the purpose of this paper was to share successful organizational partnership experiences from the perspectives of researchers, we recognize the importance of also examining the partners’ experience and recommendations. Our team is currently conducting a study with youth, parents, clinicians, and organizational leaders to examine their experiences of partnering with *CanChild*. Therefore, this paper may be limited in scope as it was focused on providing information about organizational partnerships in a childhood disability context from researchers to other researchers and organizations. It is important to acknowledge that organizational partners might not perceive the same experiences, benefits, and lessons learned as the researchers involved in this study. Therefore, our companion study will include and compare the experiences of childhood disability research partners.

## Conclusions

In summary, researchers shared experiences of establishing partnerships with organizations from a childhood disability research centre. Through a collaborative auto-ethnography approach and subsequent reflection, we shared lessons-learned and provided recommendations that other researchers can utilize when forming, evolving, and maintaining partnerships with organizations. The key ingredients of successful organizational partnerships were identified to be reciprocity, infrastructure, and relationship-building. Future work in this area should understand factors that contribute to the longevity of organizational partnerships and to understand the first-hand experiences of organizational partners.

## Data Availability

Not applicable.
